# Supplementary material for: Saccharomyces boulardii (CNCM I-745) improves intestinal damage in sepsis by remodeling intestinal flora structure
Source: Front Cell Infect Microbiol. 2025 Jul 31;15:1602792. doi: 10.3389/fcimb.2025.1602792 (PMC12350329; doi:10.3389/fcimb.2025.1602792)
Supplement: Supplementary file 1 [file DataSheet1.zip › summary/summary/8_Advanced_analyse/2_PICRUSt2_result/KEGG/Group.CW4_vs_AW4/PICRUSt2_KO_Group.CW4_vs_AW4_diff.pdf]

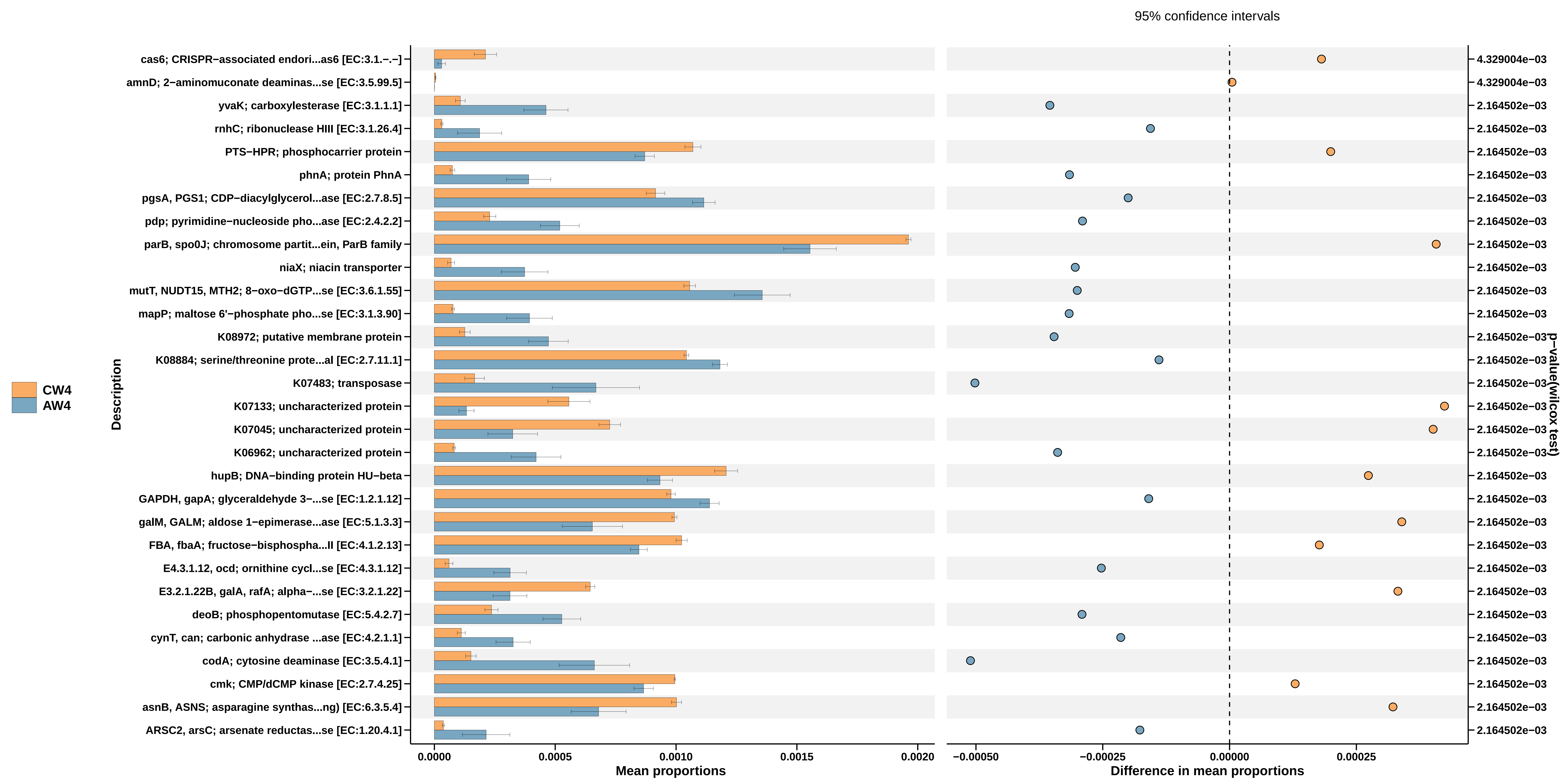
95% confidence intervals
CW4
AW4
Description
cas6; CRISPR−associated endori...as6 [EC:3.1.−.−]
amnD; 2−aminomuconate deaminas...se [EC:3.5.99.5]
yvaK; carboxylesterase [EC:3.1.1.1]
rnhC; ribonuclease HIII [EC:3.1.26.4]
PTS−HPR; phosphocarrier protein
phnA; protein PhnA
pgsA, PGS1; CDP−diacylglycerol...ase [EC:2.7.8.5]
pdp; pyrimidine−nucleoside pho...ase [EC:2.4.2.2]
parB, spo0J; chromosome partit...ein, ParB family
niaX; niacin transporter
mutT, NUDT15, MTH2; 8−oxo−dGTP...se [EC:3.6.1.55]
mapP; maltose 6'−phosphate pho...se [EC:3.1.3.90]
K08972; putative membrane protein
K08884; serine/threonine prote...al [EC:2.7.11.1]
K07483; transposase
K07133; uncharacterized protein
K07045; uncharacterized protein
K06962; uncharacterized protein
hupB; DNA−binding protein HU−beta
GAPDH, gapA; glyceraldehyde 3−...se [EC:1.2.1.12]
galM, GALM; aldose 1−epimerase...ase [EC:5.1.3.3]
FBA, fbaA; fructose−bisphospha...II [EC:4.1.2.13]
E4.3.1.12, ocd; ornithine cycl...se [EC:4.3.1.12]
E3.2.1.22B, galA, rafA; alpha−...se [EC:3.2.1.22]
deoB; phosphopentomutase [EC:5.4.2.7]
cynT, can; carbonic anhydrase ...ase [EC:4.2.1.1]
codA; cytosine deaminase [EC:3.5.4.1]
cmk; CMP/dCMP kinase [EC:2.7.4.25]
asnB, ASNS; asparagine synthas...ng) [EC:6.3.5.4]
ARSC2, arsC; arsenate reductas...se [EC:1.20.4.1]
0.0000
0.0005
0.0010
0.0015
0.0020
Mean proportions
−0.00050
−0.00025
0.00000
0.00025
Difference in mean proportions
p−value(wilcox test)
4.329004e−03
4.329004e−03
2.164502e−03
2.164502e−03
2.164502e−03
2.164502e−03
2.164502e−03
2.164502e−03
2.164502e−03
2.164502e−03
2.164502e−03
2.164502e−03
2.164502e−03
2.164502e−03
2.164502e−03
2.164502e−03
2.164502e−03
2.164502e−03
2.164502e−03
2.164502e−03
2.164502e−03
2.164502e−03
2.164502e−03
2.164502e−03
2.164502e−03
2.164502e−03
2.164502e−03
2.164502e−03
2.164502e−03
2.164502e−03
